# Genome-Wide Identification of Genes Probably Relevant to the Uniqueness of Tea Plant (*Camellia sinensis*) and Its Cultivars

**DOI:** 10.1155/2015/527054

**Published:** 2015-10-12

**Authors:** Yan Wei, Wang Jing, Zhou Youxiang, Zhao Mingming, Gong Yan, Ding Hua, Peng Lijun, Hu Dingjin

**Affiliations:** Institute of Quality Standard and Testing Technology for Agro-Products, Hubei Academy of Agricultural Sciences, Wuhan 430064, China

## Abstract

Tea (*Camellia sinensis*) is a popular beverage all over the world and a number of studies have focused on the genetic uniqueness of tea and its cultivars. However, molecular mechanisms underlying these phenomena are largely undefined. In this report, based on expression data available from public databases, we performed a series of analyses to identify genes probably relevant to the uniqueness of *C. sinensis* and two of its cultivars (LJ43 and ZH2). Evolutionary analyses showed that the evolutionary rates of genes involved in the pathways were not significantly different among *C. sinensis*, *C. oleifera*, and *C. azalea*. Interestingly, a number of gene families, including genes involved in the pathways synthesizing iconic secondary metabolites of tea plant, were significantly upregulated, expressed in *C. sinensis* (LJ43) when compared to *C. azalea*, and this may partially explain its higher content of flavonoid, theanine, and caffeine. Further investigation showed that nonsynonymous mutations may partially contribute to the differences between the two cultivars of *C. sinensis*, such as the chlorina and higher contents of amino acids in ZH2. Genes identified as candidates are probably relevant to the uniqueness of *C. sinensis* and its cultivars should be good candidates for subsequent functional analyses and marker-assisted breeding.

## 1. Introduction

Tea (*Camellia sinensis*) is one of the most popular beverages in the world. It belongs to the genus* Camellia* (Theaceae: Ericales) and originated from East Asia [1]. The cultivation of* C. sinensis* probably started more than 2,000 years ago [1]. The extensive secondary metabolites in tea leaves, including polyphenols, theanine, and volatile oils, are good for people's health [2]. Nowadays, many cultivars of* C. sinensis*, such as Longjing 43 (LJ43), Zhonghuang 1 (ZH1), and Zhonghuang 2 (ZH2), are cultivated extensively in China. In addition to* C. sinensis*, the genus* Camellia* includes many other species of great value, such as* C. oleifera* and* C. azalea*.* C. oleifera* has been cultivated for thousands of years in China. It is a kind of small tree with multiple trunks and branches. Its seeds can be pressed to yield edible tea oil, more than 80% of which is monounsaturated fat [3]. Unlike the aforementioned two species,* C. azalea* is very precious and was discovered in Guangdong Province decades ago. This is a very beautiful plant with great ornamental value.

A transcriptome is all the transcripts expressed in one or a population of cells at a certain time. With the advent of next-generation sequencing technology, a great number of transcriptomes, especially those from nonmodel species, have been reported. Given their important economic and ornamental values, transcriptomes of the above three* Camellia* species have been reported and some of their unique characteristics were identified. Specifically, Shi et al. described the transcriptome of* C. sinensis* and identified candidate genes related to natural product pathways that are important to tea quality, such as genes involved in flavonoid, theanine, and caffeine biosynthesis pathways [4]; Wang et al. compared the biochemical and transcriptomic differences between LJ43 and ZH2 to uncover mechanisms underlying their phenotypic differences [5]; and Xia et al. characterized the transcriptome from tender shoots, young leaves, flower buds, and flowers of* C. oleifera *and detected many genes potentially related to lipid metabolism [6]. Despite this body of work, the molecular events underlying the uniqueness of* C. sinensis* and its cultivars remain largely undefined.

Identifying coding regions harboring mutations probably relevant to the uniqueness of a species or a cultivar has attracted the interests of many biologists for decades. Generally, nonsynonymous substitutions are harmful for their carriers and will be eliminated rapidly. However, a small number of nonsynonymous substitutions will benefit their carriers and genes harboring those mutations are termed positively selected genes. When a population extends its range or is moved by human activity to a new environment with environmental factors that are different to the original one, a series of genes may be subject to positive selection and may ultimately result in a new species. Comparing the numbers of nonsynonymous (*dN*) and synonymous (*dS*) substitutions per site is often used for diagnosing the extent and direction of selection on sequence evolution, with the ratio *dN*/*dS* >1, =1, and <1 denoting positive evolution, neutral evolution, and purifying evolution, respectively [7, 8]. Church et al. demonstrated that using likelihood-based variable selection models is feasible for comparing sequence pairs [9]. In fact, many studies have reported analyses identifying positively selected genes for several different species [10–12]. It seems highly likely that a number of genes may be subject to positive selection during the speciation of* C. sinensis*. Moreover, during the cultivation of* C. sinensis*, many important agronomical genes may be subject to artificial selection and may therefore result in a new cultivar.

In this report, based on the transcriptomes reported for the three species of the genus* Camellia*, we identified genes potentially relevant to the uniqueness of* C. sinensis*. Furthermore, candidate genes probably relevant to the divergence of LJ43 and ZH2 were also detected. Our results should be important for understanding the uniqueness of* C. sinensis* and its cultivars and provide hints for subsequent breeding.

## 2. Materials and Methods

### 2.1. Data Acquisition and Filtering

Paired-end Illumina short reads generated for the floral bud transcriptome of* C. azalea* (PRJNA257896) [13] and 454 reads for transcriptomes of tender shoots, leaves, flowers, and flower buds of* C. oleifera *[6] generated by Xia et al. (PRJNA239933), along with 454 reads of the new shoot transcriptome generated by Wang et al. for four* C. sinensis* strains (PRJNA223181) [14], were downloaded from the Sequence Read Archive at the National Center for Biotechnology Information (SRA, http://www.ncbi.nlm.nih.gov/sra/). In addition, paired-end Illumina short reads of the transcriptome of the two cultivars of* C. sinensis*, LJ43 (PRJNA261659, PRJNA240661, and PRJNA79643) and ZH2 (PRJNA261659), were downloaded from SRA.

Quality controls were implemented using NGS QC Toolkit_v2.3.3 with default settings [15]. Low quality bases that reside in short reads generated using Illumina and Roche 454 platforms were filtered by scripts included in this package. Single-end 454 reads were further processed using Seqclean (http://sourceforge.net/projects/seqclean/files/latest/download) to trim the vector sequences included in UniVec (ftp://ftp.ncbi.nih.gov/pub/UniVec/).

### 2.2. *De Novo* Assembly

A* de novo* assembly based on paired-end Illumina short reads was performed using Trinity [16] with default settings for* C. azalea*. For the assembly of 454 reads of the other two species, the iAssembler package was used, which employs MIRA (http://sourceforge.net/projects/mira-assembler) and CAP3 [17] and can assemble large-scale ESTs into consensus sequences with significantly higher accuracy [18]. For each species, contigs shorter than 200 bp were discarded in the ensuing analyses. For the* de novo* transcriptome assembly for each species, TransDecoder (http://sourceforge.net/projects/transdecoder/) was used for predicting the probable open reading frames.

### 2.3. Identification of Orthologous Genes and Alignment

Tentative orthologs among the three species were predicted using a transitive Reciprocal Best Hits (RBH) approach implemented in the Ortholuge pipeline [19] with default settings except the *e*-value for blastn being set to 1*e* − 9. For each ortholog group, we compared each nucleotide sequence with the corresponding protein sequence predicted for* C. oleifera* using Genewise [20] and used a customized Perl script to extract the matched coding regions and generate the proper alignment format for the subsequent PAML [8] analyses. We excluded alignments with premature stop codons or those shorter than 30 codons after deleting the gaps.

### 2.4. Evolutionary Analyses

The evolutionary rates (*K*_*a*_, *K*_*s*_, and *K*_*a*_/*K*_*s*_) for each ortholog group and each separate species in the genus* Camellia* were calculated using CODEML under the branch-free model (model = *b*). To test for selection acting on* C. sinensis*, we used CODEML's branch-site models (model = 2, NSsites = 2), which allowed *dN*/*dS* to vary among codon sites and across branches of the phylogeny. By setting the branch leading to* C. sinensis* as the foreground branch, we compared a selection model that allowed a class of codons on that branch to have *dN*/*dS* > 1 (Model A2, fix_omega = 0, omega = 1.5) with a neutral model that constrained this additional class of sites to have *dN*/*dS* = 1 (Model A1, fix_omega = 1, omega = 1). A likelihood ratio test (LRT) with *χ* ^2^ approximation was used to determine the significance of difference between the nested models. All these analyses were implemented twice with different starting omega values to check for convergence. Alignments of positive results were checked and adjusted manually and subject to analyses once again to reduce the possibility of false positives.

### 2.5. Mutations between LJ43 and ZH2 and Experimental Verification

All Illumina paired-end reads of LJ43 and ZH2 were used for identifying mutation sites potentially relevant to their divergence. For these two cultivars, all reads were mapped back to the nonredundant transcriptome using BWA (-n 0.005 -k 5) [21], and all duplicate reads were removed using the MarkDuplicates program from the software package Picard (http://broadinstitute.github.io/picard/). Reads with minimum mapping quality < 20 were removed using SAMtools [22], and then a synchronized file was generated using the program mpileup2sync in the software package PoPoolation2 [23]. The synchronized file listed allele frequencies for every population at every base in the nonredundant transcriptome in a concise format. Sites with base frequencies more than five in LJ43 but absent from ZH2 were identified as unique mutations and were compared with the predicted transcript structure to decide if they were located in the coding regions and changed the encoding amino acids.

Due to the fact that RNA-Seq may produce false positives, mutation sites residing in some genes and probably related to the phenotype differentiation were selected and subject to experimental verification. Briefly, total RNAs from the leaves of LJ43 and ZH2 were extracted with Trizol Reagent Kit (Invitrogen, Madison, USA) and were reverse transcribed into cDNA using the M-MLV RTase cDNA synthesis kit (Takara, Dalian, China); sequences involved in the pathway “Porphyrin and chlorophyll metabolism” harboring nonsynonymous mutations were selected and amplified using gene-specific primers (Table 1). Sequences of the amplified productions were bidirectionally sequenced using an ABI 3730 Model DNA sequencer (Shanghai Sangon, China) and submitted to Genbank.

### 2.6. Pathway Analysis

KEGG pathways were assigned to all the ortholog groups using the KOBAS software [24]. After that, the *K*_*a*_ values for each pathway with more than fifteen genes assigned were compared between* C. sinensis* and the other two species separately using the binomial test. Contigs with mutation sites that encoded different amino acids between LJ43 and ZH2 or were identified as positively selected genes in* C. sinensis* were individually subject to pathway enrichment analysis using the KOBAS software.

### 2.7. Differentially Expressed Gene Families between* C. sinensis* and* C. azalea*

The identification of differentially expressed genes between species is blocked by the accurate assignment of ortholog relationships, especially for nonmodel species for which whole genome sequences are unavailable. To understand the pivotal genes probably relevant to the uniqueness of* C. sinensis* such as the high content of flavonoid, theanine, and caffeine, we employed a strategy used for the identification of differentially expressed gene families cross species [25]. Briefly, the proteome of* Arabidopsis thaliana* was downloaded from Ensembl Plants (http://plants.ensembl.org/) and clustered as gene families using CD-HIT [26]. The sequence identity threshold was set to 0.6 and a representative sequence for each gene family was selected. Then, short Illumina reads for LJ43 and* C. azalea* were mapped back to the* de novo* assembled transcriptomes of* C. sinensis* and* C. azalea *separately, using bowtie [27] with default settings. After that, each sequence of the transcriptomes of the two species was uniquely mapped to the representative of each gene family, and the number of mapped short reads was accumulated if two or more sequences were mapped to the same gene family for each species. Finally, edgeR [28] was used to identify the differentially expressed gene families between the two species, and the method of Benjamini and Hochberg was used to adjust *p* values for multiple comparisons [29]. Changes in expression patterns of gene families including genes involved in the pathways synthesizing “flavonoid,” “theanine,” and “caffeine” were scrutinized [4].

## 3. Results and Discussion

With* de novo* assembly methods, we obtained 246,972, 103,002, and 141,099 sequences for* C. azalea*,* C. sinensis*, and* C. oleifera*, respectively. The number of sequences obtained for* C. azalea* was similar to that reported previously [13]. However, using the Newbler software, other researchers obtained 60,479 and 120,425 sequences for* C. sinensis* and* C. oleifera*, respectively [6, 14]. The significantly greater number of sequences we obtained for these two species may be because Newbler performs best for restoring full-length transcripts [30, 31], but iAssembler can identify incorrectly assembled contigs and should be more conservative [18]. Moreover, the greater number of sequences for* C. azalea* was much more than the other two species and may result from the different sequencing platforms and assemblers, since short reads for* C. azalea* were generated by using Illumina sequencing technology and the long reads for the other two species were generated by 454 pyrosequencing.

**Figure 1:**
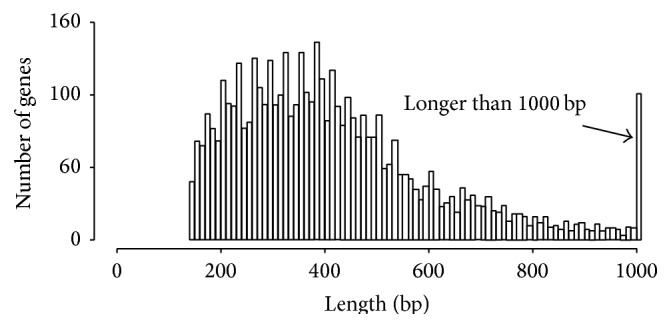
Length distribution of sequence alignments for the ortholog groups among* C. azalea*,* C. sinensis*, and* C. oleifera*.

Using the transitive RBH method, 8,787 one-one-one ortholog groups were identified and 4,617 groups were selected for subsequent evolutionary analyses. Distribution of alignment length for each ortholog group reveals that most groups are shorter than 600 bp. This may be because sequences used in the current analyses were generated by RNA-Seq and have a bias for shorter sequence reads (Figure 1).

Using a likelihood method and the binomial test, we found that the evolutionary rates of genes involved in metabolic pathways were not significantly different among the three species (data not shown). The likelihood method identified a total of 97 sequences as positively selected genes in* C. sinensis*, and pathways that these sequences may participate in are shown in Supplemental Table S1 (in the Supplementary Material available online at http://dx.doi.org/10.1155/2015/527054). The pathways are mainly related to the metabolism of carbohydrates, lipids, amino acids, and some secondary metabolites, such as “glycolysis/gluconeogenesis,” “steroid biosynthesis,” “fatty acid degradation,” “arginine and proline metabolism,” “histidine metabolism,” and “butanoate metabolism.” However, genes participating in pathways synthesizing important secondary metabolites of the species, such as “flavonoid,” “theanine,” and “caffeine,” were not identified in the current analyses. These results may be because positively selected genes involved in these pathways were not included in the original dataset and the conservatism of the method in the condition of few sequences [32]. Further investigations employing the transcriptomes of more species may address this issue.

Changes of expression patterns of some pivotal genes may also contribute to the uniqueness of* C. sinensis*. To test this hypothesis, differentially expressed gene families between* C. sinensis* (LJ43) and* C. azalea* were identified. Specifically, profiles of the genes that participate in the synthesis of the three important secondary metabolites in* C. sinensis *are shown in Table 2. We found that most gene families including the genes that participate in the pathways synthesizing “flavonoid,” “theanine,” and “caffeine” were significantly upregulated and expressed in* C. sinensis*. In contrast, the significance of gene families including those genes that were downregulated in* C. sinensis* was not so notable, except the family including the gene “leucoanthocyanidin reductase.” Thus, we suggest that the uniqueness of* C. sinensis* may result from the upregulation of some pivotal genes. However, the reliability of the results needs further investigation since these results are based on the comparison of expression patterns of gene families and employing the combined expression data from different tissues.

Mutation analyses found polymorphisms present between LJ43 and ZH2 for more than 10,000 sites. Further investigations showed that 3,655 mutations were located in the coding regions and 2,021 of them were nonsynonymous mutations. Pathway analysis showed that genes harboring these nonsynonymous mutations were involved in a number of pathways. In particular, the pathways “alanine, aspartate, and glutamate metabolism,” “Porphyrin and chlorophyll metabolism,” “glycine, serine, and threonine metabolism,” “valine, leucine, and isoleucine degradation,” and “flavonoid biosynthesis” were well represented (Supplemental Table S2). Gene ontology analysis showed that genes with those nonsynonymous mutations were significantly enriched in GO terms “chloroplast part” and “chloroplast stroma” (Supplemental Table S2). To validate the mutations identified using high-throughput analyses, the sequences involved in the pathway “Porphyrin and chlorophyll metabolism” were selected and subjected to experimental verification. Bidirectional sequencing confirmed seven of the eleven nonsynonymous sites residing in these genes (Table 1). Biochemical analyses revealed that contents of free amino acids and flavonoid are different in the yellow-leaf tea cultivar ZH2 and normal cultivar LJ43 [5]. Our study suggests that nonsynonymous mutations residing in the coding regions of some genes may also take part in the formation of differences between the two cultivars, in addition to the differential expression of some other genes [5].

## Supplementary Material

Supplemental Table S1 KEGG pathways assigned to the positively-selected candidates identified in C. sinensis.
Supplemental Table S2 KEGG pathways and GO terms assigned to the genes harboring nonsynonymous mutations in ZH2 compared with LJ43.

## Figures and Tables

**Table 1:** Profiles of the validation of genes harboring nonsynonymous mutations in ZH2 compared with LJ43.

Serial number	Genbank accession number (LJ43)	Genbank accession number (ZH2)	Description	Mutation site	LJ43	ZH2	Confirmed or not	Forward primer	Reverse primer
UN010458	KT427374	KT427366	Uroporphyrinogen decarboxylase	1301	A	G	No	CGCAGATTTGAGACTGGTTG	TGGCCAATCAAGTGAAGATG
UN010458	KT427374	KT427366	Uroporphyrinogen decarboxylase	1310	A	G	Yes	CGCAGATTTGAGACTGGTTG	TGGCCAATCAAGTGAAGATG
UN010458	KT427374	KT427366	Uroporphyrinogen decarboxylase	1421	A	G	Yes	CGCAGATTTGAGACTGGTTG	TGGCCAATCAAGTGAAGATG
UN014682	KT427373	KT427365	Ferrochelatase 1	1745	T	C	No	ACTTTCTCCAAGGCTGCTC	CCAACACGGGTACTAACG
UN016015	KT427372	KT427364	Glutamyl-tRNA reductase 1	674	G	C	Yes	AAGGCCAACTCAACAGAAGC	AGTTGGGCAAGGAGTCACTG
UN020635	KT427371	KT427363	Cytochrome c oxidase assembly protein COX15	446	G	C	Yes	GATCCCTCCATCGTCATCAT	CATGCAGCAGAAGCAAAAAG
UN028022	KT427367	KT427359	Chlorophyllase 1	346	T	C	Yes	TTACAGGAGCAATAGTAGGTT	CCATAGTAGAGGTGGAAAGA
UN031492	KT427368	KT427360	Chlorophyllase 2	176	A	G	Yes	GAATGTAAACCGCCAAGT	CATCCAAACAAGCCCTTA
UN043046	KT427369	KT427361	Chlorophyll(ide) b reductase NOL	183	A	T	No	TCTTCTGTTGTCTGGCGCTT	TCGATTCCTCCTAGCAACCAA
UN047188	KT427370	KT427362	Glutamyl-tRNA synthetase	128	T	A	No	CAAAAGCAAAAGCACCCAACC	GAACCCACCACCATACTCGC
UN047188	KT427370	KT427362	Glutamyl-tRNA synthetase	93	G	C	Yes	CAAAAGCAAAAGCACCCAACC	GAACCCACCACCATACTCGC

**Table 2:** Profiles of the expression patterns of gene families (*C. sinensis* (LJ43) versus *C. azalea*) including genes involved in the pathways synthesizing flavonoid, theanine, and caffeine.

Secondary metabolic pathway	Gene name	Number of members of the family	Fold change (log⁡2)	*p* value	*p* adj.
Flavonoid biosynthesis	Phenylalanine ammonia lyase	4	1.73	0.01	0.04
	Cinnamate 4-hydroxylase	1	0.82	0.21	0.40
	4-Coumarate CoA ligase	3	−0.02	0.98	0.99
	Flavanone 3-hydroxylase	1	−0.06	0.92	0.97
	Flavonol synthase	3	1.98	0.003	0.02
	Leucoanthocyanidin reductase	2	−2.35	0.0006	0.004
	Glutamine synthetase	6	1.15	0.08	0.20

Theanine biosynthesis	Gamma-glutamyl transpeptidase	1	−0.20	0.76	0.87
	Alanine aminotransferase	1	2.06	0.002	0.01
	S-Adenosylmethionine decarboxylase	3	−0.10	0.88	0.94

Caffeine biosynthesis	GMP synthase	1	0.65	0.32	0.53
	5′-Nucleotidase	1	1.56	0.02	0.07
	Adenylosuccinate synthase	1	2.29	0.0008	0.005
	AMP deaminase	1	0.002	1	1
	S-Adenosylmethionine synthase	4	0.76	0.25	0.44
